# Prevalence of chronic kidney disease in patients with chronic obstructive pulmonary disease: a systematic review and meta-analysis

**DOI:** 10.1186/s12890-016-0315-0

**Published:** 2016-11-24

**Authors:** Swarna Gaddam, Sameer K. Gunukula, James W. Lohr, Pradeep Arora

**Affiliations:** 1 Division of Nephrology at VAMC, Buffalo, NY USA; 2 Department of Medicine, SUNY, Buffalo, NY USA; 3 Division of Nephrology at VAMC, Richmond, VA USA; 4 Department of Medicine, Virginia Commonwealth University, Richmond, VA USA

**Keywords:** Chronic obstructive pulmonary disease, Emphysema, Chronic bronchitis, Chronic kidney disease, Comorbidity, Glomerular filtration rate

## Abstract

**Background:**

The incidence and prevalence of chronic kidney disease (CKD) continue to rise worldwide. Increasing age, diabetes, hypertension, and cigarette smoking are well-recognized risk factors for CKD. Chronic obstructive pulmonary disease (COPD) is characterized by chronic airway inflammation leading to airway obstruction and parenchymal lung destruction. Due to some of the common pathogenic mechanisms, COPD has been associated with increased prevalence of CKD.

**Methods:**

Systematic review of medical literature reporting the incidence and prevalence of CKD in patients with COPD using the Cochrane Collaboration Methodology, and conduct meta-analysis to study the cumulative effect of the eligible studies. We searched Medline via Ovid, PubMed, EMBASE and ISI Web of Science databases from 1950 through May, 2016. We included prospective and retrospective observational studies that reported the prevalence of CKD in patients with COPD.

**Results:**

Our search resulted in 19 eligible studies of which 9 have been included in the meta-analysis. The definition of CKD was uniform across all the studies included in analysis. COPD was found to be associated with CKD in the included epidemiological studies conducted in many countries. Our meta-analysis showed that COPD was found to be associated with a significantly increased prevalence of CKD (Odds Ratio [OR] = 2.20; 95% Confidence Interval [CI] 1.83, 2.65). Study limitations: Studies included are observational studies. However, given the nature of our research question there is no possibility to perform a randomized control trial.

**Conclusions:**

Patients with COPD have increased odds of developing CKD. Future research should investigate the pathophysiological mechanism behind this association, which may lead to better outcomes.

**Electronic supplementary material:**

The online version of this article (doi:10.1186/s12890-016-0315-0) contains supplementary material, which is available to authorized users.

## Background

Chronic kidney disease (CKD) is a major public health problem in the United States, with rising incidence and prevalence of kidney failure, with poor outcomes and high cost. There is an even higher prevalence of earlier stages of CKD. According to the Third National Health and Nutrition Examination Survey (NHANES III), it was estimated that 6.2 million people (3% of total US population) above the age of 12 years had a serum creatinine above 1.5 mg/dl and 8 million people had a glomerular filtration rate (GFR) <60 ml/min/1.73 m^2^ The majority of these people are greater than 65 years of age (5.9 million).

Diabetes, hypertension and cardiovascular disease are associated with greater prevalence of CKD [1, 2]. In addition, CKD has a complicated interrelationship with these diseases. As per United States Renal Data System (USRDS data), prevalence of stage 3 CKD has been increasing. Although the prevalence of hypertension (HTN) did not rise, the incidence of diabetes mellitus (DM) has increased 4 fold from 1980 to 2014 [3]. Studies have reported that CKD is an independent risk factor for cardiovascular disease [4]. In recent years, additional causes of CKD have been recognized such as unrecovered acute kidney injury (AKI) [5, 6] and use of proton pump inhibitors (PPIs) [7, 8], In this manuscript we analyze the association of CKD with COPD using the limited data available… Several studies have identified COPD as part of a systemic inflammatory syndrome [9–12] and reported on the association of comorbidities like lung cancer [13], osteoporosis [14], progression of atherosclerosis [15], and CKD. This systematic review was performed to assess the association of COPD with CKD.

## Methods

### Eligibility criteria

We included prospective and retrospective observational studies that reported the prevalence of CKD in patients with COPD when compared to those without COPD. Most of the studies established the diagnosis of COPD using Spirometry or ICD-9 codes obtained from their medical records while others were based on history or physician diagnosed COPD. Prevalence of CKD in the majority of the studies was reported based on eGFR obtained from laboratory data. We only included studies that reported data on adult populations.

### Search strategy

In January 2016, we electronically searched PubMed, Medline (1950 onwards; access via Ovid), EMBASE (all years; access via Ovid) and Web of Science using a detailed search strategy with search terms described below and in Additional file 1. After initial detailed discussion of the aim of the study, the search strategy was outlined by the authors. Search terms used for COPD were: ‘COPD’, ‘chronic obstructive pulmonary disease’, ‘emphysema’, ‘chronic bronchitis’. Search terms used for CKD were: ‘chronic kidney disease’, ‘CKD’, ‘ESRD’, ‘end stage renal disease’, ‘renal insufficiency’, ‘renal failure’.

Inclusion criteria: We included all prospective and retrospective observational studies reporting the prevalence of CKD in patients with COPD compared to those without COPD; there were no restrictions on language of publication.

Exclusion criteria: We excluded studies that did not report the association of CKD with COPD; those that were not focused on the association of CKD with COPD; studies that did not have appropriate methodological study design of comparison groups; studies with data which was incompletely recorded; and those involving pediatric populations.

We reviewed keywords and related studies. From using the selected studies, we proceeded further in the literature search looking for “related articles” in PubMed. References of the included studies were manually searched to ensure inclusion of all related articles.

### Selection process

Two reviewers independently reviewed the titles and abstracts of the citations resulting from the search using a standardized screening guide. Full text was obtained for the articles which were thought to be eligible by at least one of the reviewers.. Each reviewer reviewed these full texts independently to judge their eligibility to be included in our review. Disparities between the two reviewers about which studies should be included were discussed and resolved by a third reviewer.

### Data abstraction

Data was independently extracted from the included studies by two reviewers (SG and SGK) using a standardized and pilot-tested form for data abstraction. Any differences in extracted data were discussed by the reviewers, and if not resolved, by discussion with a third reviewer. The pilot-tested form included study design, method used to diagnose COPD and CKD, population, methodological characteristics of the study, and the reported results. We recorded the effect measures derived from the regression models that adjusted for the maximum number of covariates. We rated the overall quality of evidence for each outcome using the Grading of Recommendations Assessment, Development and Evaluation (GRADE) approach [16].

### Data analysis

The kappa statistic was calculated to determine the agreement between the two reviewers of the full texts of the studies for inclusion eligibility. Meta-analysis was performed from the studies that reported the prevalence/incidence of CKD in patients with COPD compared to patients without COPD. In studies that did not report actual number of events, other available data (such as percentages) was used to calculate the number of events for the evaluated outcomes. For each outcome, we pooled the odds ratios (OR) of eligible studies using the generic inverse variance and the random effects model in Review Manager Version 5.3. The random effects model was used as the included studies evaluated different patient populations. We measured homogeneity across study results using the I^2^ statistic. We examined possible publication bias using inverted funnel plots.

## Results

Our extensive electronic database search of databases in January, 2016 resulted in 7583 articles. A flow diagram with detailed outline of literature search is provided in Additional file 2. After detailed review of the articles, our literature search resulted in 17 studies related to the topic of our research. Manual search for related articles helped identify an additional study that was recently published and did not have a PubMed ID [17]. Another study was published in April 2016 while we were in the process of submission for publication [18]. As a result, we included 19 articles related to our topic of interest.

Nine of these studies reported the prevalence of CKD in patients with COPD compared to those without COPD [17–26]. We reported a detailed review of these studies with data extraction in Table 1 and we included the results reported in these studies in a meta-analysis to estimate the cumulative effect. The remaining 10 studies did not meet inclusion criteria for meta-analysis [27–35]. A detailed review of these articles is reported in Table 2. Eight of these 10 studies have been excluded since they are longitudinal studies that reported the prevalence of CKD in a cohort of patients with COPD [18, 27–30, 32–34]. One of the studies has been excluded due to the study design and since the data could not be used for analysis [31]. One other study was excluded due to use of non-standardized method to assess the prevalence of CKD and therefore did not meet criteria to be included in the systematic review [35]. The reviewers had very good agreement on study eligibility (kappa = 0.983). Using GRADE approach [16], the quality of evidence from the studies included in our review was found to be moderate to low.

**Table 1:** Systematic review of 9 studies reporting prevalence of CKD in patients with COPD compared to controls; included in meta-analysis

Study	Population	COPD diagnosis method & Definition of CKD	Methodological features	Results
Baty et al.; 2013 [8] Study design: Population based case-control study Funding: Takeda Pharma AG, Switzerland	Setting & period: All hospitalizations in Switzerland between 2002 and 2010 COPD group: 340, 948 patients, 64% males, median age 73 years Non-COPD group: 340,948 patients, 64% males, median age 73 years	Diagnosis of COPD: Based on ICD-10 codes CKD definition: Based on ICD-10 code Blinding of outcome adjudicator: not reported	Selection bias: none Information bias: objective outcome evaluation: no; standardized CKD risk measurement: no Confounding: no Matching: yes. Adjustment in analysis: yes Confounding variables: no Loss to follow up: none	4.39% of patients with COPD had Chronic kidney disease (ICD 10 code, N188) compared to 2.13% of patients without COPD (*p* < 0.001) 4.64% of patients with COPD had Chronic kidney disease unspecified (ICD 10 code, N189) compared to 2.25% of patients without COPD (*p* < 0.001)
Gjerde et al.; 2011 [9] Study design: Case-control study Funding: The Foundation for Respiratory Research, Center for Clinical Research, Bergen	Setting & period: Patients aged 40-76 years with COPD were recruited from health institutions in Hordaland County in Western Norway, where as those without COPD were recruited among former participants from a general population survey in Hordaland County; between 2006 and 2007 COPD group: 433 patients, 59.6% male Non-COPD group: 233 patients	Diagnosis of COPD: using Spirometry CKD definition: eGFR <60 Blinding of outcome adjudicator: not reported	Selection bias: yes, voluntarily included, not random Information bias: objective outcome evaluation: yes; standardized CKD risk measurement: yes Confounding: yes Matching: no. Adjustment in analysis: yes Confounding variables: no Loss to follow up: none	Prevalence of undiagnosed renal failure in the COPD patients was 6.9%, significantly higher than among the subjects without COPD (*p* < 0.001)
Incalzi et al.; 2010 [10] Study design: Case-control study Funding: not reported	Setting & period: Participants aged 65 years and older were recruited from pulmonary medicine outpatient facilities in University of Palermo, Italy COPD group: 356 patients Non-COPD group: 290 patients	Diagnosis of COPD: Spirometry CKD definition: eGFR < 60 using MDRD equation Blinding of outcome adjudicator: not reported	Selection bias: no Information bias: objective outcome evaluation: yes; standardized CKD risk measurement: yes Confounding: no Matching: yes (age) Adjustment in analysis: yes Confounding variables: no Loss to follow up: none	Overall prevalence of Chronic renal failure was 43.0% in COPD group and 23.4% in non-COPD group (*p* < 0.001) Logistic regression analysis revealed significant association between COPD and concealed chronic renal failure (OR: 2.19; CI: 1.17-4.12) and overt chronic renal failure (OR: 1.94; CI: 1.01-4.66)
Joo et al.; 2012 [11] Study design: Cross-sectional Survey Funding: Grant of Korea Healthcare Technology R&D project	Setting & period: Database of the fourth Korean Health and Nutrition Examination Survey with a nationally representative sample, during 2008. Aged ≥ 40 years COPD group: 354 patients, 67.2% male, mean age 64.6 years Non-COPD group: 1823 patients, 36.9% male, mean age 54.4 years	Diagnosis of COPD: Spirometry, FEV1/FVC < 0.7 CKD definition: patients’ awareness of CKD diagnosis was surveyed Blinding of outcome adjudicator: not reported	Selection bias: no Information bias: objective outcome evaluation: yes, for COPD diagnosis only; standardized CKD risk measurement: no Confounding: yes Matching: no. Adjustment in analysis: yes Confounding variables: gender, mean age Loss to follow up: none	0.6% of patients in COPD group had Chronic renal failure compared to 0.4% in non-COPD group (*p* = 0.41, not statistically significant)
Mapel et al.; 2013 [12] Study design: retrospective case-control cohort analysis Funding: grant from Pfizer Pharmaceuticals Inc.	Setting & period: patients aged 40 years or older seen in 4 hospitals and a network of outpatient clinics of Lovelace Health Systems (LHS) in New Mexico, USA during the study period 2005-2008 COPD group: 2284 patients of LHS aged 40 or more with COPD and have at least 2 outpatient clinic visits or one hospitalization and enrolled with LHS for at least 12 months during the study period; 47.5% men; mean age 70.3 +/- 9.8 yrs Non-COPD group: 5959 randomly selected patients without a diagnosis of COPD and be of same age and gender	Diagnosis of COPD: ICD-9 diagnosis code of COPD CKD definition: ICD-9 codes and abnormal renal function tests Blinding of outcome adjudicator: not reported	Selection bias: none Information bias: objective outcome evaluation: yes; standardized CKD risk measurement: yes Confounding: no Matching: yes (age, gender). Adjustment in analysis: yes Confounding variables: no Loss to follow up: none	Chronic renal failure was more than three times more prevalent among COPD patients (2.89%) than among controls (0.79%) (*p* < 0.001)
Nagorni-Obradovic; 2014 [13] Study design: cross sectional study (case-control analysis) Funding: Ministry of Education and Science of the Republic of Serbia	Setting & period: 10,013 nationally representative sample of adults aged 40 years or older who participated in multipurpose health survey of population of Serbia in 2006 COPD group: 653 patients, 46.6% male, mean age 62.8 years (SD: 12.4) Non-COPD group: 9.360 patients, 54.4% male, mean age 59.3 years (SD: 12.2)	Diagnosis of COPD: Self-reported history of chronic bronchitis and emphysema CKD definition: Self-reported history of chronic renal disease Blinding of outcome adjudicator: N/A	Selection bias: no Information bias: objective outcome evaluation: no; standardized CKD risk measurement: no Confounding: no Matching: no Adjustment in analysis: yes (age, gender, educational level, smoking) Confounding variables: no Loss to follow up: n/a	20.6% of COPD patients reported having a diagnosis of chronic renal failure compared to 9.3% of non-COPD patients (*p* < 0.01)
Schnell et al., 2012 [14] Study design: cross-sectional study (case-control analysis) Funding: Johns Hopkins, NCRR and NIH	Setting & period: non-institutionalized civilians in the US aged 45 years or more who participated in the National Health and Nutrition Examination Survey (NHANES) from 1998 through 2008 COPD group: 995 patients, 39.9% males, mean age 62.7 years (CI: 61.7-63.8) Non-COPD group: 14,828 patients, 47% males, mean age 60 years (CI: 59.6-60.3)	Diagnosis of COPD: positive response in NHANES questions to either chronic bronchitis or emphysema with negative response to current asthma CKD definition: NHANES question with positive response to eGFR < 60 as calculated using MDRD equation Blinding of outcome adjudicator: N/A	Selection bias: no Information bias: objective outcome evaluation: no; standardized CKD risk measurement: yes Confounding: no Matching: no Adjustment in analysis: yes Confounding variables: none Loss to follow up: n/a	16.2% of patients with physician diagnosed COPD reported having low eGFR, compared to 10.5% of patients without physician diagnosed COPD (*p* < 0.0001)
Van Gestel et al.; 2009 [15] Study design: cohort study Funding: none	Setting & period: 3358 patients who underwent elective vascular surgery or lower limb arterial reconstruction surgeries between January 1990 to December 2006 COPD group: 1310 patients Non-COPD group: 2048 patients	Diagnosis of COPD: post bronchodilator pulmonary function test CKD definition: based on calculated eGFR <60 estimated using MDRD equation Blinding of outcome adjudicator: not reported	Selection bias: yes, convenience sample Information bias: objective outcome evaluation: yes; standardized CKD risk measurement: yes Confounding: yes. Matching: No. Adjustment in analysis: yes Confounding variables: age, gender, type of surgery, current smoking, previous heart failure, hypertension, Diabetes, hyperlipidemia Loss to follow up: none	COPD was associated with a higher risk of prevalent CKD even after adjustment for confounding variables – OR: 1.22 (1.03 – 1.44) (*p* = 0.03) A borderline significant relationship was observed for mild COPD while moderate COPD was independently associated with CKD. No significant association was found between severe COPD and CKD
Yoshizawa et al.; 2015 [6] Study design: retrospective case-control cohort analysis Funding: none	Setting & period: outpatient clinic visits of Kanamecho Hospital, Tokyo, Japan for the study period of May 2011 to April 2012 COPD group: 108 stable COPD patients; 83.3% males; mean age 74.3 ± 7.1 year. Non-COPD group: 73 patients of the same outpatient practice; 49.3% males; mean age 71.8 ± 7.3 years	Diagnosis of COPD: spirometry reading of FEV1/FVC less than 70% after inhalation of a bronchodilator, and severity of obstruction judged according to the Global Initiative for Chronic Obstructive Lung Disease (GOLD) criteria CKD definition: eGFR less than 60 mL/min/1.73 m2 as per calculation based on serum Creatinine and serum Cystatin levels separately Blinding of outcome adjudicator: not reported	Selection bias: no Information bias: objective outcome evaluation: yes; standardized CKD risk measurement: yes Confounding: yes. Matching: No. Adjustment in analysis: yes Confounding variables: age, gender, BMI, hypertension, Diabetes, hyperlipidemia Loss to follow up: none	Prevalence of CKD (using Se Cr for calculation of eGFR) was significantly higher in COPD group - OR: 4.91 (1.94 – 12.46) (*p* = 0.0004) Prevalence of CKD (using Se Cys for calculation of eGFR) was significantly higher in COPD group - OR: 6.30 (2.99 – 13.26) (*p* < 0.0001)

**Table 2:** Systematic review of 10 studies reporting prevalence of CKD in patients with COPD; excluded from meta-analysis

Study	Population	COPD diagnosis method & Definition of CKD	Methodological features	Results
Almagro et al., 2002 [17] Study design: prospective cohort study Funding: not reported	Setting & period: patients hospitalized to an acute-care hospital in Barcelona (Spain) for acute exacerbation of COPD, between October 1996 and May 1997 Patient group: 135 patients, 96% male, median age 72.2 ± 9.25 years	Diagnosis of COPD: Spirometry CKD definition: not defined, diagnosis information obtained from Charlson index	Selection bias: yes, patients admitted with COPD exacerbation Information bias: Objective outcome evaluation: no; standardized CKD risk measurement: no	4.4% of the patients are reported to have renal failure
Almagro et al., 2009 [18] Study design: Cross-sectional, multi-center study Funding:	Setting & period: patients admitted with COPD exacerbation to any of the participating 26 hospital centers throughout Spain, consecutively between January 1, 2007 and December 31, 2008 Patient group: 398 patients, 89% male, mean age of 73.7 years	Diagnosis of COPD: Spirometry CKD definition: not defined, comorbidity information obtained from Charlson index and an ad hoc questionnaire	Selection bias: patients admitted with COPD exacerbation Information bias: Objective outcome evaluation: no; standardized CKD risk measurement: no	6.5% of patients are reported to have moderate kidney failure
Almagro et al.; 2012 [16] Study design: Longitudinal, observational, multi-center study Funding: provided by Chiesi España	Setting & period: Patients hospitalized for COPD exacerbation to 70 ED and internal medicine services in Spain between October 2009 and October 2010 Patient group: 606 patients, 89.9% male, median age 72.6 years (range, 41-94)	Diagnosis of COPD: Spirometry CKD definition: not defined, diagnosis information obtained using Charlson index and a questionnaire	Selection bias: yes, patients admitted with COPD exacerbation Information bias: Objective outcome evaluation: no; standardized CKD risk measurement: no	15.5% of patients are reported to have Kidney disease with serum creatinine <3 0.7% of patients are reported to have Kidney disease with serum creatinine >3
Antonelli Incalzi et al., 1997 [19] Study design: Retrospective cohort study Funding: not reported	Setting & period: Consecutive patients discharged from Catholic University in Rome between the years 1980 and 1990, after an acute exacerbation of COPD Patient group: 270 patients, 83% male, mean age 67 ± 9 (SD) years	Diagnosis of COPD: Spirometry CKD definition: not defined, obtained from Charlson’s index	Selection bias: patients likely with severe COPD Information bias: Objective outcome evaluation: no; standardized CKD risk measurement: no	6.6% of patients were noted to have chronic renal failure Death in these patients was predicted by several variables including chronic renal failure (HR 1.79; 95% CI 1.05–3.02)
Chen et al.; 2016 [7] Study design: Case-Cohort study Funding: Ministry of Science of Technology, Taiwan	Setting & period: Patients aged 40 years or older who had inpatient hospitalization between 1998 and 2008 with Longitudinal Health Insurance Database (LHID) 2000 as the case group COPD group: 7,739 patients, 67.5% males, mean age 71.7 years Non-COPD group: 15,478 patients, 67.5% males, mean age 71.7 years	Diagnosis of COPD: Based on hospitalization for COPD CKD definition: Clinical diagnosis Blinding of outcome adjudicator: not reported	Selection bias: none Information bias: objective outcome evaluation: yes; standardized CKD risk measurement: yes Confounding: no Matching: yes. Adjustment in analysis: yes Confounding variables: yes; age, gender, first diagnosis of COPD Loss to follow up: none	Overall incidence of CKD was higher in COPD group than in non-COPD group. The adjusted hazard ratio of case was 1.61 (*P* <0.0001) times that of control.
Ford, E S.; 2015 [20] Study design: retrospective case-control study Funding: None	Setting & period: 5711 American men and women aged 40 to 79 years who participated in the Third National Health and Nutrition Examination Survey (NHANES III) during the term 1988 through 1994 and followed through 2006 COPD group: 1390 participants Non-COPD group: 4321 participants	Diagnosis of COPD: spirometry CKD definition: eGFR calculation using the Chronic Kidney Disease Epidemiology Collaboration equations Blinding of outcome adjudicator: not reported	Selection bias: no Information bias: objective outcome evaluation: yes; standardized CKD risk measurement: yes Confounding: no Matching: yes. Adjustment in analysis: yes Confounding variables: no Loss to follow up: none	The rates of incidence or prevalence of CKD was not reported. Comparative data on mean eGFR values in COPD group and Non-COPD group was reported. Adjusted mean levels of eGFR were significantly lower in adults with moderate-severe COPD (87.7 mL/min/1.73 m2) than in adults with normal lung function (89.6 mL/min/1.73 m2) (*p* = 0.015)
García-Olmos et al., 2013 [21] Study design: Observational, cross-sectional study Funding: CDTI/Ministry of Science and Innovation	Setting & period: practice population allocated to 129 Family Physicians, conducted in a health area of the Madrid Patient group: 3,183 patients, 76% male, mean age of 71.41 ± 11.50 years	Diagnosis of COPD: from clinical history in EMR CKD definition: not defined, obtained from EMR	Selection bias: not validated COPD diagnostic method Information bias: Objective outcome evaluation: no; standardized CKD risk measurement: no	6.34% of patients have chronic renal failure
Marti et al., 2005 [22] Study design: Retrospective cohort study Funding: In part by grant from Fundacio ‘noma’Catalana de Pneumologia and by Red Respira-ISCIII-RTIC-03/11	Setting & period: patients with COPD initiating LTOT >15 h/day during 1992–1999 in a tertiary teaching hospital (Vall d’Hebron Hospital, Barcelona, Spain) Patient group: 128 patients, 98.4% male, mean age ± SD 68.9 ± 9.7 years	Diagnosis of COPD: PFTs CKD definition: not defined, assessed using Charlson index	Selection bias: yes, COPD patients only on long term O2 therapy Information bias: Objective outcome evaluation: no; standardized CKD risk measurement: no	1.6% of patients are reported to have renal disease
Terzano et al., 2010 [23] Study design: Prospective longitudinal study Funding:	Setting & period: Consecutive COPD patients admitted to four hospitals in Italy for acute exacerbation from 1999 to 2000, and followed up until December 2007 Patient group: 288 patients, 78.8% male, mean age 69.2 years (SD ± 6.4)	Diagnosis of COPD: standardized CKD definition: not defined, assessed using Charlson index	Selection bias: yes, patients admitted for acute exacerbation Information bias: Objective outcome evaluation: no; standardized CKD risk measurement: no	26.3% of patients are reported to have chronic renal failure
Van Manen et al.; 2001 [24] Study design: case control study Funding: Boehringer Ingelheim NL supplied materials and personnel for performing lung function testing	Setting & period: Adults aged 40 years or more who visited outpatient practices in urban and suburban regions of western part of Netherlands from October 1996 through June 1997 COPD group: 290 patients (male 64.1%; mean age 65.8 years) Non-COPD group: 421 patients (male 41.1%; mean age 65.9 years)	Diagnosis of COPD: Pulmonary function tests CKD definition: not reported Blinding of outcome adjudicator: not reported	Selection bias: no Information bias: objective outcome evaluation: yes; standardized CKD risk measurement: no Confounding: no Matching: no Adjustment in analysis: yes Confounding variables: no Loss to follow up: none	The study population was surveyed to estimate the prevalence of a set of 23 diseases in patients with COPD compared to patients without COPD. Self-reported renal disease was included in general and no specifications on chronic kidney disease or renal failure was surveyed. Renal disease was reported 0.3% in patients with COPD compared to 0.2% in non-COPD patients

**Fig. 1:**
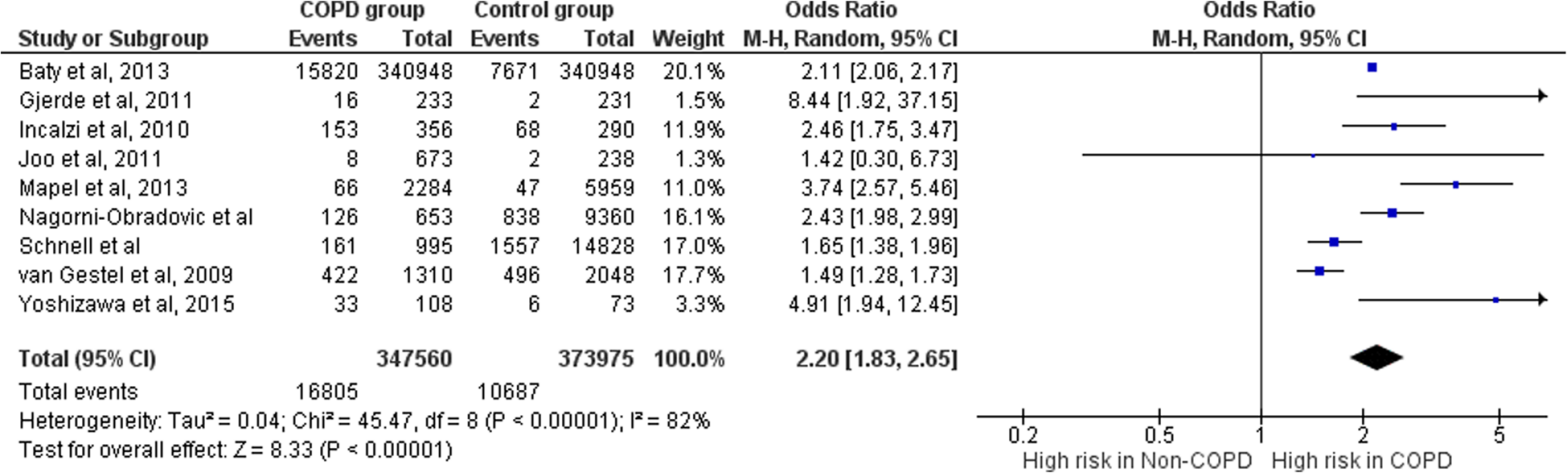
Meta- analysis to assess the cumulative prevalence of CKD in patients with COPD when compared to control groups

**Fig. 2:**
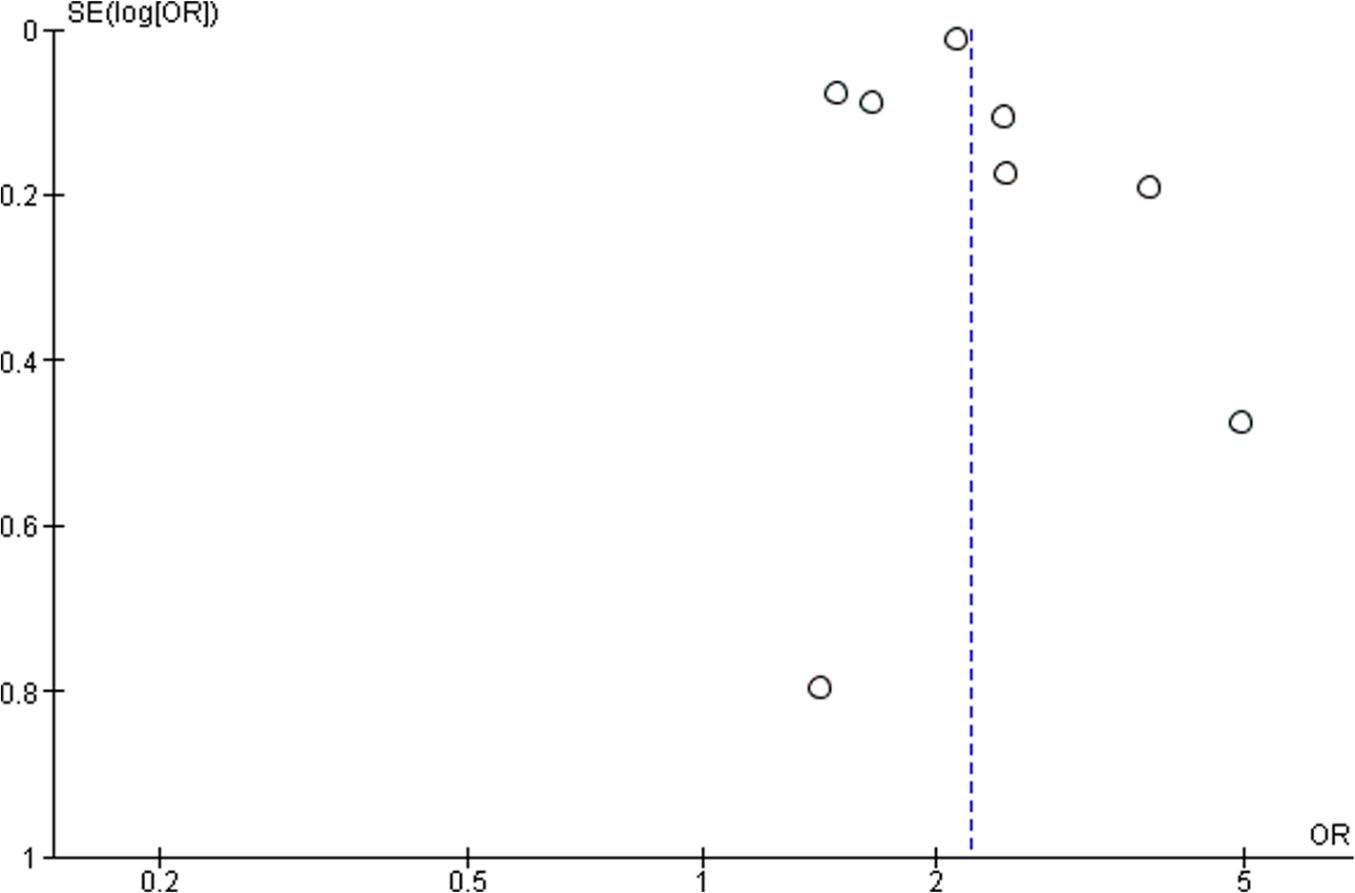
Funnel plot to assess for publication bias among the studies included in meta-analysis

Results from meta-analysis (Fig. 1) showed statistically significant higher prevalence of CKD among COPD patients compared to controls without COPD; OR 2.20 [95% CI: 1.83, 2.65]. I^2^ value of 82% indicates minimal heterogeneity among the studies included in the analysis. Funnel plot of the included studies revealed lack of publication bias (Fig. 2).

The study periods of almost all the studies were in the last 2 decades except one study [30]. Three of the 9 studies included in meta-analysis utilized nationally representative samples in their respective countries [22, 24, 25]. Only 2 studies reported data on hospitalized patients [19, 26] while the remaining 4 studies included patients from outpatient settings [17, 20, 21, 23]. Of the 10 studies that were excluded from the meta-analysis, six studies reported data on hospitalized patients [18, 27–30, 34]

Patient population included was aged 40 years or older. Studies included in the meta-analysis had similar gender representation with male participant rates reported from 40% to 65% except one study that reported 83% male participation [17]. Nine of the 10 studies that were not included in the meta-analysis but were included in the systematic review reported predominant male participation ranging from 80% to 98% [18, 27–34].

Of the studies included in meta-analysis, only one study analyzed prevalence of CKD in COPD and control groups based on gender [24]. Van Gestel et al. analyzed the association between prevalence of CKD and severity of COPD [26]. Therefore sub-group analysis could not be performed for prevalence based on gender and severity of COPD.

The reported diagnostic methods and definitions for COPD and CKD were uniform across all the included studies. The value of eGFR reported under laboratory data was used to define CKD in most of these studies. Matching was performed in control group selection in 3 of the 9 studies included in the meta-analysis [19, 21, 23]. All the included studies performed adjustment in analysis for the identified confounding variables.

## Discussion

Our meta-analysis validated previously published results showing a significant increase in prevalence of CKD in patients with COPD compared to patients without COPD. To our knowledge, this is the first meta-analysis conducted on this topic.

Advancing age, diabetes, hypertension, body mass index (BMI), and cigarette smoking have previously been identified as risk factors for new-onset kidney disease [36]. Advancing age, history of asthma, severe respiratory problems in childhood, passive smoking, and exposure to biomass fuel for heating were identified as risk factors for COPD in never-smokers whereas increasing age, history of asthma, and severe respiratory problems in childhood, increasing lifetime exposure to cigarette smoking were identified as independent risk factors for development of COPD in ever-smokers [37]. Many studies have reported on the high prevalence of CKD in COPD patients across different populations and our meta-analysis validated these previously published results. Moreover, all the studies included in our analysis adjusted for co-variates including age, gender, BMI, and smoking status and this allowed for drawing a conclusion on the independent association of CKD with COPD.

The mechanism by which COPD potentiates the development of CKD remains unclear. Several hypotheses have been put forward. It might be related to the fact that COPD is mainly a disease of the elderly population who have comorbidities such as DM, HTN and CAD, known risk factors associated with CKD. COPD has been associated with systemic inflammation [9–12]. Pro-inflammatory cytokines, especially tumor necrosis factor-alpha (TNF-α), play an important role in inflammation [38, 39], and have been shown to increase endothelial inflammation and atherosclerosis. This inflammation is also potentially related to development of diabetes, muscle wasting, and kidney disease. In a meta-analysis of observational studies, COPD was associated with increased serum concentration of several inflammatory mediators [40]. This association can be explained in part by smoking.

COPD is associated with microalbuminuria and in hypoxemic and hypercapnic patients effective renal flow was found to be reduced. These changes may be reflective of increased renin-angiotensin system activity seen in COPD patients. In the Multi-Ethnic Study of Atherosclerosis cohort, Harris et al. found an inverse relation between FEV1 and FVC with urinary albumin excretion and urine albumin to urine creatinine ratio [41]. This finding suggests that systemic microvascular injury may contribute to development of CKD in COPD patients.

Medical management of COPD may contribute to the development of CKD. Mapel et al. [23] showed that COPD patients were more likely to be on potentially nephrotoxic medications than controls. This includes recurrent use of antibiotics, as well as PPIs and certain cardiovascular drugs. Our study highlights the high prevalence of CKD in COPD patients and draws attention to the clinical implications.

Our study has a few limitations. First and foremost, we included observational studies in this systematic review and meta-analysis. Observational studies, inherently, depict associations and aid in hypothesis-making but do not establish cause and effect relationships. Additionally, we could not perform subgroup analysis to estimate the differential prevalence of CKD in relation to the severity of COPD that would have otherwise contributed to establishing causal relationship. Another limitation is that although 18 studies were identified to be relevant, only 9 studies could be included in our meta-analysis owing to differences in the study designs and reported results. However, funnel plot analysis did not reveal any publication bias.

## Conclusions

In conclusion, results from our systematic review and meta-analysis strongly support the association of increased prevalence of CKD in patients with COPD. Implications for future research include a need for studies to further investigate the pathophysiological mechanisms in COPD patients that lead to a higher incidence of CKD in these patients. Results from these studies may then be applied to improve the treatment of COPD, reducing the incidence of CKD in COPD patients and thereafter decrease their morbidity and mortality.

## Additional files

Additional file 1: Literature search strategy in Medline via Ovid. (DOCX 13 kb)
Additional file 2: Study flow diagram. (DOCX 47 kb)

## Abbreviations

AKI: Acute kidney injury
BMI: Body mass index
CI: Confidence interval
CKD: Chronic kidney disease
COPD: Chronic obstructive pulmonary disease
eGFR: Estimated glomerular filtration rate
ESRD: End stage renal disease
FEV1: Forced expiratory volume in 1 second
FVC: Forced vital capacity
GFR: Glomerular filtration rate
NHANES III: Third National Health and Nutrition Study
OR: Odds ratio
PPI: Proton pump inhibitor

## References

[1] Haroun MK, Jaar BG, Hoffman SC, Comstock GW, Klag MJ, Coresh J (2003). Risk factors for chronic kidney disease: a prospective study of 23,534 men and women in Washington County Maryland. J Am Soc Nephrol.

[2] Perneger TV, Brancati FL, Whelton PK, Klag MJ (1994). End-stage renal disease attributable to diabetes mellitus. Ann Intern Med.

[3] Snively CS, Gutierrez C (2004). Chronic kidney disease: prevention and treatment of common complications. Am Fam Physician.

[4] Weiner DE, Tighiouart H, Amin MG, Stark PC, MacLeod B, Griffith JL (2004). Chronic kidney disease as a risk factor for cardiovascular disease and all-cause mortality: A pooled analysis of community-based studies. J Am Soc Nephrol.

[5] Wu VC, Huang TM, Lai CF, Shiao CC, Lin YF, Chu TS (2011). Acute-on-chronic kidney injury at hospital discharge is associated with long-term dialysis and mortality. Kidney Int.

[6] Hsu RK, Hsu CY (2016). The role of acute kidney injury in chronic kidney disease. Semin Nephrol.

[7] Lazarus B, Chen Y, Wilson FP, Sang Y, Chang AR, Coresh J, Grams ME (2016). Proton pump inhibitor use and the risk of chronic kidney disease. JAMA Intern Med.

[8] Arora P, Gupta A, Golzy M, Patel N, Carter RL, Jalal K, Lohr JW (2016). Proton pump inhibitors are associated with increased risk of development of chronic kidney disease. BMC Nephrol.

[9] Barnes PJ (2014). Cellular and molecular mechanisms of chronic obstructive pulmonary disease. Clin Chest Med.

[10] Comer DM, Kidney JC, Ennis M, Elborn JS (2013). Airway epithelial cell apoptosis and inflammation in COPD, smokers and nonsmokers. Eur Respir J.

[11] Kryvenko V (2013). Biomarkers of systemic inflammation, oxidative stress and their interactions in patients with combined flow of chronic obstructive pulmonary disease and arterial hypertension. Georgian Med.

[12] Miller J, Edwards LD, Agusti A, Bakke P, Calverley PM, Celli B (2013). Comorbidity, systemic inflammation and outcomes in the ECLIPSE cohort. Respir Med.

[13] Houghton AM (2013). Mechanistic links between COPD and lung cancer. Nat Rev Cancer.

[14] Inoue D, Watanabe R, Okazaki R (2016). COPD and osteoporosis: links, risks, and treatment challenges. Int J Chron Obstruct Pulmon Dis.

[15] Savransky V, Nanayakkara A, Li J, Bevans S, Smith PL, Rodriguez A, Polotsky VY (2007). Chronic intermittent hypoxia induces atherosclerosis. Am J Respir Crit Care Med.

[16] Atkins D, Best D, Briss PA, Eccles M, Falck-Ytter Y, Flottorp S (2004). Grading quality of evidence and strength of recommendations. BMJ.

[17] Yoshizawa T, Okada K, Furuichi S, Ishiguro T, Yoshizawa A, Akahoshi T (2015). Prevalence of chronic kidney diseases in patients with chronic obstructive pulmonary disease: assessment based on glomerular filtration rate estimated from creatinine and cystatin C levels. Int J Chron Obstruct Pulmon Dis..

[18] Chen CY, Liao KM (2016). Chronic obstructive pulmonary disease is associated with risk of chronic kidney disease: a nationwide case-cohort study. Sci Rep.

[19] Baty F, Putora PM, Isenring B, Blum T, Brutsche M (2013). Comorbidities and burden of COPD: a population based case-control study. PLoS One.

[20] Gjerde B, Bakke PS, Ueland T, Hardie JA, Eagan TM (2012). The prevalence of undiagnosed renal failure in a cohort of COPD patients in western Norway. Respir Med.

[21] Incalzi RA, Corsonello A, Pedone C, Battaglia S, Paglino G, Bellia V (2010). Chronic renal failure: a neglected comorbidity of COPD. Chest.

[22] Joo H, Park J, Lee SD, Oh YM (2012). Comorbidities of chronic obstructive pulmonary disease in Koreans: a population-based study. J Korean Med Sci.

[23] Mapel DW, Marton JP (2013). Prevalence of renal and hepatobiliary disease, laboratory abnormalities, and potentially toxic medication exposures among persons with COPD. Int J Chron Obstruct Pulmon Dis..

[24] Nagorni-Obradovic LM, Vukovic DS (2014). The prevalence of COPD co-morbidities in Serbia: results of a national survey. NPJ Prim Care Respir Med..

[25] Schnell K, Weiss CO, Lee T, Krishnan JA, Leff B, Wolff JL (2012). The prevalence of clinically-relevant comorbid conditions in patients with physician-diagnosed COPD: a cross-sectional study using data from NHANES 1999-2008. BMC Polm.

[26] van Gestel YR, Chonchol M, Hoeks SE, Welten GM, Stam H, Mertens FW (2009). Association between chronic obstructive pulmonary disease and chronic kidney disease in vascular surgery patients. Nephrol Dial Transplant.

[27] Almagro P, Cabrera FJ, Diez J, Boixeda R, Alonso Ortiz MB, Murio C (2012). Comorbidities and short-term prognosis in patients hospitalized for acute exacerbation of COPD: the EPOC en Servicios de medicina interna (ESMI) study. Chest.

[28] Almagro P, Calbo E, Ochoa de Echaguen A, Barreiro B, Quintana S, Heredia JL (2002). Mortality after hospitalization for COPD. Chest.

[29] Almagro P, Lopez Garcia F, Cabrera F, Montero L, Morchon D, Diez J (2010). Comorbidity and gender-related differences in patients hospitalized for COPD. The ECCO study. Respir Med..

[30] Antonelli Incalzi R, Fuso L, De Rosa M, Forastiere F, Rapiti E, Nardecchia B (1997). Co-morbidity contributes to predict mortality of patients with chronic obstructive pulmonary disease. Eur Respir J.

[31] Ford ES (2015). Urinary albumin-creatinine ratio, estimated glomerular filtration rate, and all-cause mortality among US adults with obstructive lung function. Chest.

[32] Garcia-Olmos L, Alberquilla A, Ayala V, Garcia-Sagredo P, Morales L, Carmona M (2013). Comorbidity in patients with chronic obstructive pulmonary disease in family practice: a cross sectional study. BMC Fam Pract.

[33] Marti S, Munoz X, Rios J, Morell F, Ferrer J (2006). Body weight and comorbidity predict mortality in COPD patients treated with oxygen therapy. Eur Respir J.

[34] Terzano C, Conti V, Di Stefano F, Petroianni A, Ceccarelli D, Graziani E (2010). Comorbidity, hospitalization, and mortality in COPD: results from a longitudinal study. Lung.

[35] van Manen JG, Bindels PJ, IJzermans CJ, van der Zee JS, Bottema BJ, Schade E (2001). Prevalence of comorbidity in patients with a chronic airway obstruction and controls over the age of 40. J Clin Epidemiol.

[36] Fox CS, Larson MG, Leip EP, Culleton B, Wilson PW, Levy D (2004). Predictors of new-onset kidney disease in a community-based population. JAMA.

[37] Tan WC, Sin DD, Bourbeau J, Hernandez P, Chapman KR, Cowie R (2015). Characteristics of COPD in never-smokers and ever-smokers in the general population: results from the CanCOLD study. Thorax.

[38] Barnes PJ (2009). The cytokine network in chronic obstructive pulmonary disease. Am J Respir Cell Mol Biol.

[39] Pinto-Plata VM, Mullerova H, Toso JF (2006). C-reactive protein in patients with COPD, control smokers and non-smokers. Thorax.

[40] Gan WQ, Man SF, Senthilselvan A, Sin DD (2004). Association between chronic obstructive pulmonary disease and systemic inflammation: a systematic review and a meta-analysis. Thorax.

[41] Harris B, Klein R, Jerosch-Herold M, Hoffman EA, Ahmed FS, Jacobs DR (2012). The association of systemic microvascular changes with lung function and lung density: a cross-sectional study. PLoS One.

