# Supplementary material for: Prevalence of chronic kidney disease in patients with chronic obstructive pulmonary disease: a systematic review and meta-analysis
Source: BMC Pulm Med. 2016 Nov 24;16:158. doi: 10.1186/s12890-016-0315-0 (PMC5122151; doi:10.1186/s12890-016-0315-0)
Supplement: Additional file 1: — Literature search strategy in Medline via Ovid. (DOCX 13 kb) [file 12890_2016_315_MOESM1_ESM.docx]

Additional File 1.

Literature search strategy in Medline via Ovid

| 1 | COPD or chronic obstructive pulmonary disease or emphysema or chronic bronchitis | 70424 |
| --- | --- | --- |
| 2 | Chronic kidney disease or CKD or ESRD or end stage renal disease or renal insufficiency or renal failure | 137116 |
| 3 | Humans | 15668240 |
| 4 | 1 and 2 and 3 | 829 |
| 5 | Comorbidity | 94768 |
| 6 | 1 and 3 and 5 | 2236 |
| 7 | 4 or 6 | 2901 |
