# Supplementary material for: Prevalence of chronic kidney disease in patients with chronic obstructive pulmonary disease: a systematic review and meta-analysis
Source: BMC Pulm Med. 2016 Nov 24;16:158. doi: 10.1186/s12890-016-0315-0 (PMC5122151; doi:10.1186/s12890-016-0315-0)
Supplement: Additional file 2: — Study flow diagram. (DOCX 47 kb) [file 12890_2016_315_MOESM2_ESM.docx]

Additional File 2

Study Flow Diagram

7583 total citations identified

Excluded 3082 duplicate citations

Titles of 4501 articles screened for retrieval

4291 studies not related to our topic

Abstracts of 210 articles screened screenedscreened

37 potentially eligible articles

20 studies excluded:

Inappropriate study design (13)

Outcome not of interest (4)

Incomplete data reported (3)

2 recently published papers identified through citations check

10 studies excluded from meta-analysis:

Longitudinal studies (8)

Non-standard methods for diagnosis (1)

Study design unable inappropriate (1)

9 studies included in Meta-analysis

19 studies included in Systematic Review
